# The Prevalence and Genotype Distribution of Human Papillomaviruses Among Men in Henan Province of China

**DOI:** 10.3389/fmed.2021.676401

**Published:** 2021-09-20

**Authors:** Huiling Wang, Jing Zhao, Xiaoli Liu, Wenjuan Yan, Gang Li, Youhua Yuan

**Affiliations:** ^1^Department of Clinical Laboratory, Henan Provincial People's Hospital, People's Hospital of Zhengzhou University, Zhengzhou, China; ^2^People's Hospital of Henan University, Kaifeng, China

**Keywords:** human papillomaviruses (HPV), genotype distribution, Henan province, HPV vaccination, China

## Abstract

**Background:** This paper aimed to assess the prevalence of human papillomavirus (HPV) infection and the associations of sociodemographic and behavioral characteristics with HPV in unvaccinated men in Henan Province before the mass administration of the HPV vaccine through a baseline survey.

**Methods:** Between June 2015 to June 2020, 3,690 men were tested for the HPV genotype at the Henan Provincial People's Hospital. The HPV genotype was detected by a polymerase chain reaction (PCR)-based hybridization gene chip assay.

**Results:** The overall HPV infection rate was 29.97%; The most prevalent genotypes were HPV 6 (21.76%), 11 (12.68%), 16 (8.94%), 58 (5.37%), 18 (3.41%), 84 (3.25%), 61 (3.09%), and 81 (3.09%). Low-risk HPV (LR-HPV) infection (24.91%) and single infection (17.78%) were the most prevalent forms. Age-specific HPV distribution was presented as a bimodal curve; the youngest age group (≤ 25 years) had the highest HPV infection rate (36.03%), followed by the 36–40-year-old group (33.68%). Men with Junior high school or above were more likely to have Pure-LR HPV infection. Unmarried status and smoking increased single and LR-HPV infection. Multiple lifetime sex partners and not using a condom were more likely to cause LR-HPV infection.

**Conclusions:** The data on the prevalence and HPV infection type distribution in men in Henan Province could serve as a valuable reference to guide nationwide screening. We provide a time-based estimate of the maximum impact of the HPV vaccine and critical reference measurements important for assessing the clinical benefits of HPV vaccination and the increase in non-vaccine HPV types.

## Introduction

Human papillomavirus (HPV) infection has become one of the most common sexually transmitted diseases. HPV can cause cervical intraepithelial lesions, cervical cancer, and anogenital lesions through human skin and mucosal tissue infection (1). HPV genotypes can be classified based on carcinogenicity into high-risk (HR) and low-risk (LR) genotypes (2). High-risk HPV genotypes mainly include HPV 16, 18, 31, 33, 35, 39, 45, 51, 52, 56, 58, 59, 66, 68, 83, and other genotypes mainly related to the occurrence of squamous epithelial tumors, such as cervical cancer and penile cancer (3). Low-risk genotypes mainly include HPV 6, 11, 40, 42, 43, 44, 61, 70, 72, 81, and other genotypes that promote various benign lesions such as genital condyloma acuminatum (4).

HPV prevalence and genotype distribution are different between various nations and regions (5, 6). Previous studies have mainly focused on HPV infection in women; epidemiological studies of HPV infection in men have been rare; the available data are still insufficient. Regarding HPV infection in women, it has been proposed that men play an important role as reservoirs and transmission agents. Therefore, studies are needed to outline HPV infection in men and help reduce HPV infection in women through contact with HPV-infected partners (7). This study aimed to investigate the HPV infection status in men in Henan Province to guide vaccine-based HPV prevention strategies.

## Materials and Methods

### Subjects

The study population consisted of 3,690 men (age, 20–85 years) attending a male outpatient clinic from June 2015 to June 2020 in Henan Province. A man was considered eligible to participate in the study if he met the following inclusion criteria: (1) aged 18–70 years; (2) resided in Henan Province; (3) had current or past sexual activity; (4) reported no previous diagnosis of penile cancer; (5) had not participated in an HPV vaccine clinical trial; (6) reported no prior diagnosis of human immunodeficiency virus (HIV) infection or AIDS; (7) was not currently receiving treatment for a sexually transmitted infection; (8) agreed to undergo an HPV test and participate in the present study.

### Ethical Statement

This research was approved by the Ethics Committee of Henan Provincial People's Hospital (No.2021062). All of the samples and data were collected after written informed consent was provided by the participants. The management and publication of patient information in this research was strictly in accordance with the Declaration of Helsinki, including the confidentiality and anonymity, data were de-identified before analysis.

### Specimen Collection

A single cytobrush was used to collect exfoliated cells from different penile areas: the dorsal and ventral area of the penile shaft, the external and internal surface of the prepuce, coronal sulcus, glans, and distal urethra. The cells were collected in a sampling tube and stored at 4 °C until processing. The men were admonished not to wash their external genitalia the morning of the collection to increase the number of cells in the collected samples.

### HPV DNA Extraction, PCR Amplification, and Genotyping

All samples were stored in a specimen preservation medium and sent to our laboratory. DNA was obtained from disruption of the cells using the DNA Mag-Ax Kit (HybriBio Ltd.) in the automatic nucleic acid extraction instrument (HBNP-4801A HybriBio Ltd.). HPV detection and genotyping were performed using the HPV Genotyping Kit (HybriBio Ltd.), a PCR-based flow-through hybridization and gene chip system. Moreover, negative and positive quality control products were extracted throughout the entire process to make up the control group. In all, 37 type-specific probes that recognize 19 HR (16, 18, 26, 31, 33, 35, 39, 45, 51, 52, 53, 56, 58, 59, 66, 68, 70, 73, and 82) and 18 LR (6, 11, 34, 40, 42, 43, 44, 54, 55, 57, 61, 67, 69, 71, 72, 81, 83, 84) HPV genotypes were contained in the gene chip. According to the manufacturer's protocols, the PCR reaction was used to amplify the extracted DNA. The final results were obtained by colorimetric changes on the chip under direct visualization.

### Statistical Analysis

Data analysis was performed using GraphPad Prism 5 statistical analysis software (GraphPad Software, La Jolla, CA). The chi-square test was used for statistical analysis between the two groups. Odds ratios (ORs) with 95% confidence intervals (CIs) were presented using unconditional logistic regression. *P*-values were two-sided, and differences were considered statistically significant at *p* < 0.05.

## Results

### Sociodemographic and Behavioral Characteristics of the Subjects

A total of 3,690 men aged 20–85 years were included in this study. The mean age was 41.6 ± 11.3 years. The Sociodemographic and behavioral characteristics are described in Table 1. The majority of them were aged between 26 and 55. Those who attended primary school or below and Junior high school or above were roughly equal. Unmarried people were more than twice the married ones. About 60.62% of the men smoked. More than half of them had two or more sexual partners, and only 15.83% used a condom every time.

**Table 1:** Sociodemographic and behavioral characteristics in 3,690 Men.

**Variables**	**Total**	**%**
**Age/y**		
≤ 25	680	18.43%
26–55	2,920	79.13%
≥56	90	2.44%
**Education level**		
Primary school or below	1,578	42.76%
Junior high school or above	2,112	57.24%
**Marital status**		
Married	1,376	37.29%
Not married	2,314	62.71%
**Current smoker**		
NO	1,453	39.38%
YES	2,237	60.62%
**Number of sexual partners**		
0–1	1,661	45.01%
≥2	2,029	54.99%
**Frequency of condom use**		
Every time	584	15.83%
Sometimes	1,489	40.35%
Never	1,617	43.82%

### Prevalence and Genotype Distribution of HPV

**Figure 1:**
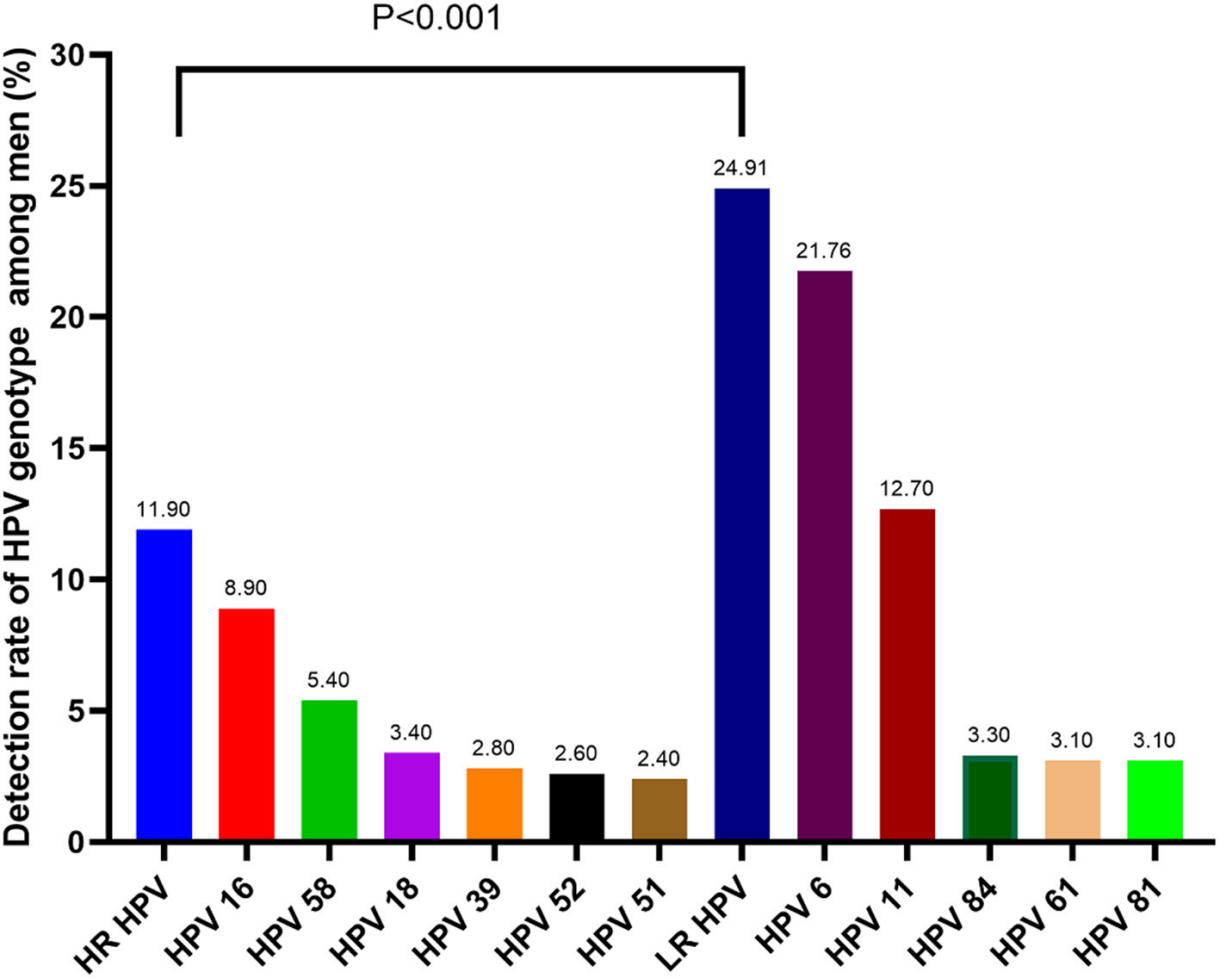
The most common HPV genotype among men (*p* < 0.001, HR-HPV *vs*. LR-HPV).

As is shown in Table 2, thirty-two of the 37 HPV genotypes that our HPV-DNA assay could detect were found, including 17 HR HPV genotypes (16, 18, 31, 33, 35, 39, 45, 51, 52, 53, 56, 58, 59, 66, 68, 73, and 82). The remaining 15 genotypes were LR HPV, including genotypes 6, 11, 34, 40, 42, 43, 44, 54, 55, 61, 67, 69, 71, 81, and 84. The overall rate of HPV infection was 29.97% (1106/3690). The most prevalent genotypes were HPV 6 (21.76%), 11 (12.68%), 16 (8.94%), 58 (5.37%), 18 (3.41%), 84 (3.25%), 61 (3.09%), and 81 (3.09%). LR HPV infection (24.91%) was the most prevalent genotype, while the HR HPV infection rate was 11.90%. Moreover, HPV 6 was the most common LR genotype, and HPV 16 was the most common HR genotype among all patients. There was a significant difference between the HR and LR genotypes of HPV. The prevalence of the HR and LR genotypes is displayed in Figure 1.

**Table 2:** Detection rate of HPV genotype among men.

**Genotype**	**HPV-positive (n)**	**Detection rate (%)**
**High risk HPV**		
HPV-16	330	8.94
HPV-18	126	3.41
HPV-31	54	1.46
HPV-33	30	0.81
HPV-35	30	0.81
HPV-39	102	2.76
HPV-45	18	0.49
HPV-51	90	2.44
HPV-52	96	2.60
HPV-53	78	2.11
HPV-56	54	1.46
HPV-58	198	5.37
HPV-59	48	1.30
HPV-66	72	1.95
HPV-68	54	1.46
HPV-73	12	0.33
HPV-82	12	0.33
Total	439	11.90
**Low risk HPV**		
HPV-6	803	21.76
HPV-11	468	12.68
HPV-34	54	1.46
HPV-40	90	2.44
HPV-42	66	1.79
HPV-43	48	1.30
HPV-44	42	1.14
HPV-54	54	1.46
HPV-55	24	0.65
HPV-61	114	3.09
HPV-67	36	0.98
HPV-69	6	0.16
HPV-71	6	0.16
HPV-81	114	3.09
HPV-83	0	0.00
HPV-84	120	3.25
Total	919	24.91

### Associations of Sociodemographic and Behavioral Characteristics With HPV

As shown in Table 3, men with Junior high school or above were more likely to have Pure-LR HPV infection (odds ratio [OR], 1.9; 95% CI, 1.4–2.5). Unmarried status increased single (OR, 1.4; 95% CI, 1.1–1.8) and LR-HPV infection. Smoking increased single (OR, 1.3; 95% CI, 1.0–1.7) and LR-HPV infection (OR, 1.6; 95% CI, 1.2–2.1); multiple lifetime sex partners and not using a condom were more likely to cause LR-HPV infection. No other significant correlations of behavioral characteristics were found.

**Table 3:** Associations of Sociodemographic and Behavioral Characteristics with the Type-specific Human Papillomavirus in 3,690 Men.

**Variables**	**Single vs. Multiple**			**Pure-LR vs. Pure-HR**			**Pure-LR vs. Mixed**		
	**Single** **No. (%)**	**Multiple** **No. (%)**	**OR** **(95%CI)**	**Pure-LR** **No. (%)**	**Pure-HR** **No. (%)**	**OR** **(95%CI)**	**Pure-LR** **No. (%)**	**Mixed** **No. (%)**	**OR** **(95%CI)**
**Age/y**									
≤ 25	139 (20.4)	106 (15.6)	1.0 (referent)	148 (21.8)	40 (5.6)	1.0 (referent)	148 (21.8)	57 (8.4)	1.0 (referent)
26–55	498 (17.1)	337 (11.5)	0.9 (0.7–1.2)	505 (17.3)	142 (4.9)	1.0 (0.7–1.5)	505 (17.3)	188 (6.4)	1.0 (0.7–1.4)
≥56	19 (21.1)	7 (7.8)	0.5 (0.2–1.2)	14 (15.6)	5 (4.6)	1.3 (0.5–3.9)	14 (15.6)	7 (7.8)	1.3 (0.5–3.4)
**Education level**									
Primary school or below	249 (15.8)	189 (12.0)	1.0 (referent)	235 (14.9)	75 (4.8)	1.0 (referent)	235 (14.9)	128 (8.1)	1.0 (referent)
Junior high school or above	407 (19.3)	261 (12.4)	0.8 (0.7–1.1)	432 (20.5)	112 (5.3)	0.8 (0.6–1.1)	432 (20.5)	124 (5.9)	1.9 (1.4–2.5)
**Marital status**									
Married	245 (17.8)	207 (15.0)	1.0 (referent)	326 (23.7)	57 (4.1)	1.0 (referent)	326 (23.7)	69 (5.0)	1.0 (referent)
Not married	411 (17.8)	243 (10.5)	1.4 (1.1–1.8)	341 (14.7)	130 (5.6)	2.1 (1.5–3.1)	341 (14.7)	183 (7.9)	2.5 (1.8–3.5)
**Current smoker**									
NO	206 (14.1)	169 (11.6)	1.0 (referent)	215 (14.8)	52 (3.6)	1.0 (referent)	215 (14.8)	108 (7.4)	1.0 (referent)
YES	450 (20.1)	281 (12.6)	1.3 (1.0–1.7)	452 (20.2)	135 (6.0)	0.8 (0.6–1.2)	452 (20.2)	144 (6.4)	1.6 (1.2–2.1)
**Number of sexual partners**									
0–1	234 (14.1)	148 (8.9)	1.0 (referent)	207 (1.5)	85 (5.1)	1.0 (referent)	207 (1.5)	90 (5.4)	1.0 (referent)
≥2	422 (20.8)	302 (14.9)	0.9 (0.7–1.1)	460 (22.7)	102 (5.1)	1.8 (1.3–2.6)	460 (22.7)	162 (8.0)	1.2 (0.9–1.7)
**Frequency of condom use**									
Every time	64 (11.0)	48 (8.2)	1.0 (referent)	56 (9.6)	25 (4.3)	1.0 (referent)	56 (9.6)	31 (5.3)	1.0 (referent)
Sometimes	215 (14.4)	145 (9.7)	0.9 (0.6–1.4)	212 (14.2)	69 (4.6)	0.7 (0.4–1.3)	212 (14.2)	79 (5.3)	1.6 (0.9–2.5)
Never	377 (23.3)	257 (15.9)	0.9 (0.6–1.4)	399 (24.7)	93 (5.8)	1.9 (1.1–3.2)	399 (24.7)	142 (8.8)	1.0 (0.8–1.4)

### Prevalence of Single and Multiple HPV Infection

**Figure 2:**
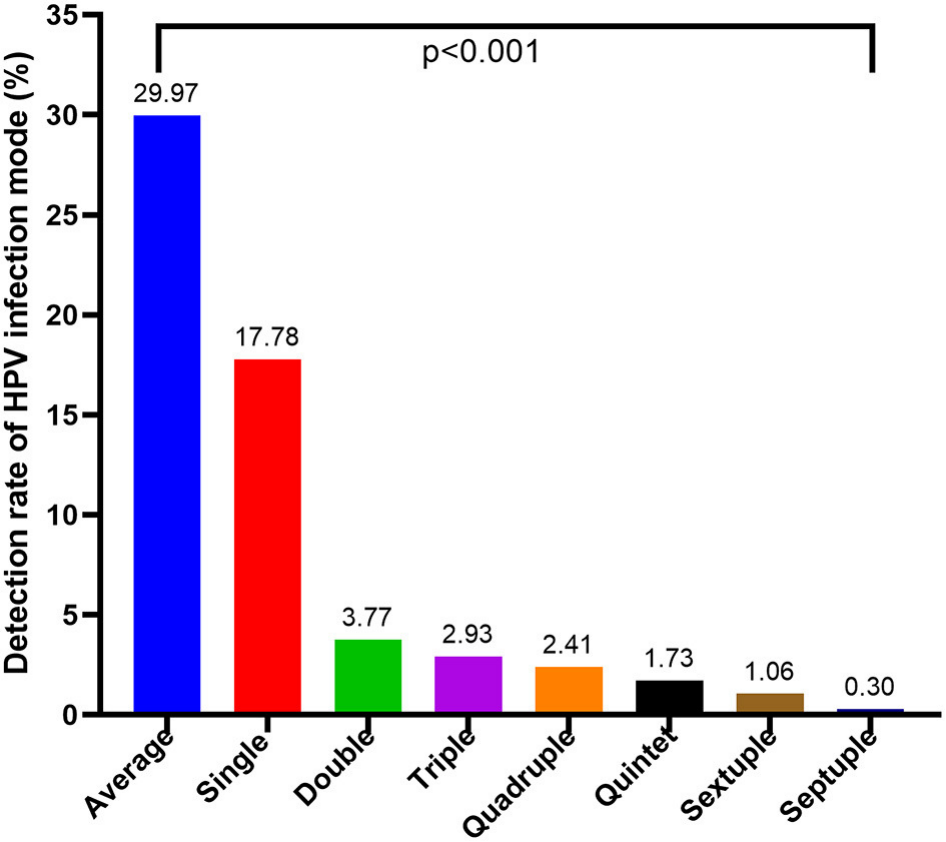
Prevalence of single and multiple HPV infections (*p* < 0.001).

**Figure 3:**
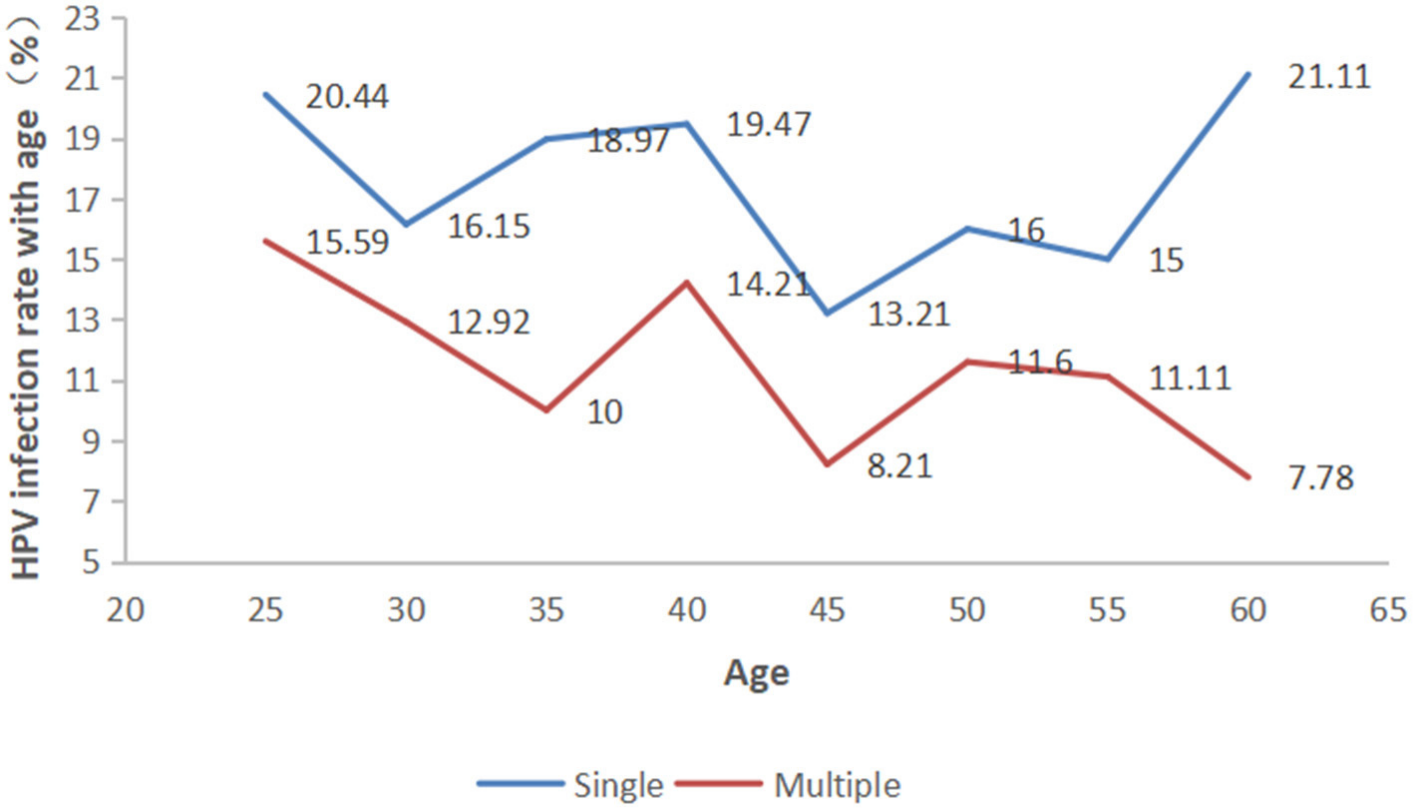
Prevalence of single and multiple HPV infections in each age group (*p* < 0.001).

As shown in Figure 2, a single HPV infection was detected in 17.78% (656/3690) of all tested men, accounting for 59.31% (656/1106) of HPV-positive men. Patients infected with more than one genotype of HPV were classified into the multiple-type infections group. Moreover, among those with multiple infections, the prevalence of HPV decreased significantly as the number of infected genotypes increased, with 139 (3.77%) patients having a double infection, 108 (2.93%) with triple infection, 89 (2.41%) quadruple infection, 64 (1.73%) quintuple infection, 39 (1.06%) sextuple infection, and 11 (0.30%) with septuple infection. The detectable rate of single-and multiple-type infections in different HPV genotypes exhibited significant differences (Supplementary Table 1, *p* < 0.001). Moreover, In the different age groups, the incidence of men infected with single- and multiple-type infections was also significantly different (Figure 3, *p* < 0.001).

### Prevalence of HPV Grouped by Age

**Figure 4:**
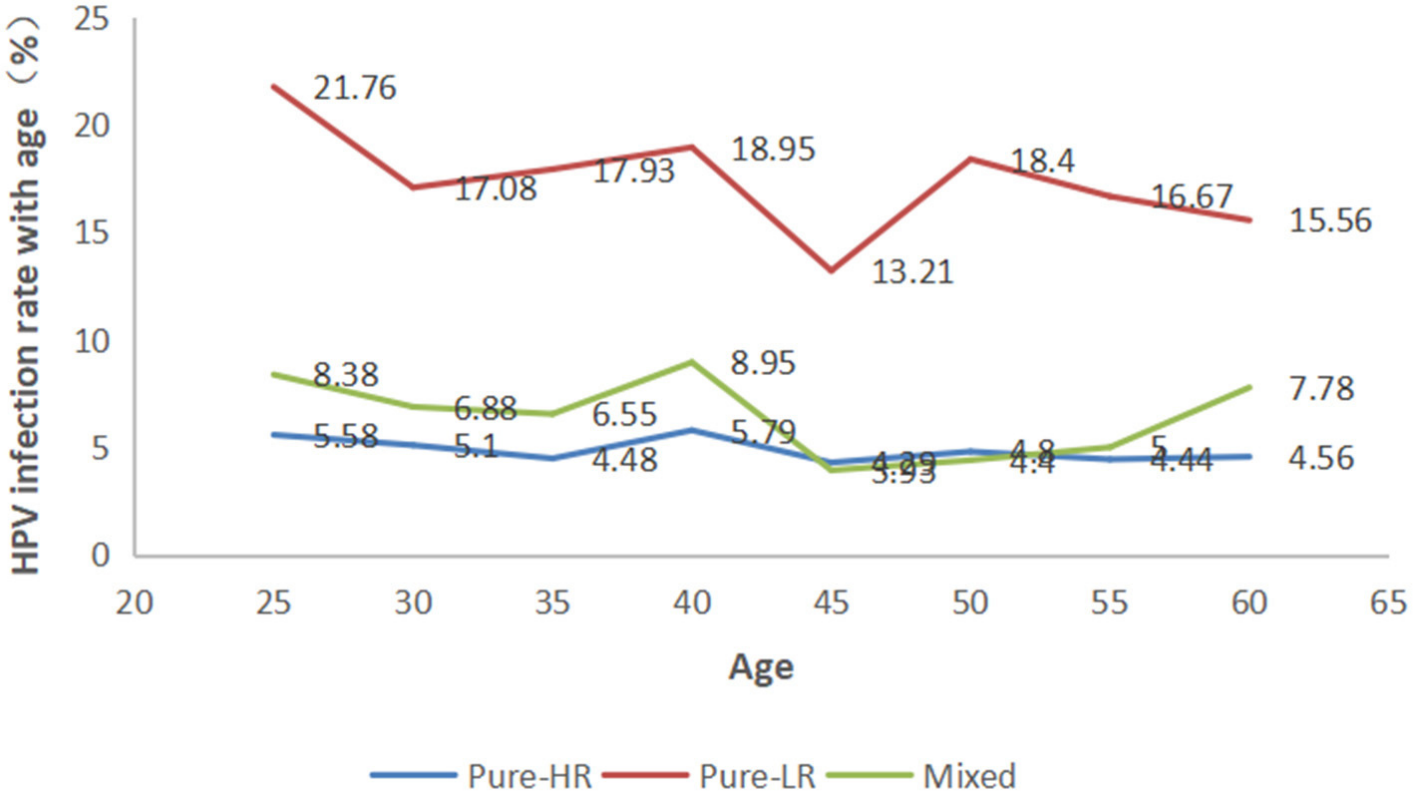
Prevalence of high-risk (HR), low-risk (LR), and mixed HPV infections in each age group.

**Figure 5:**
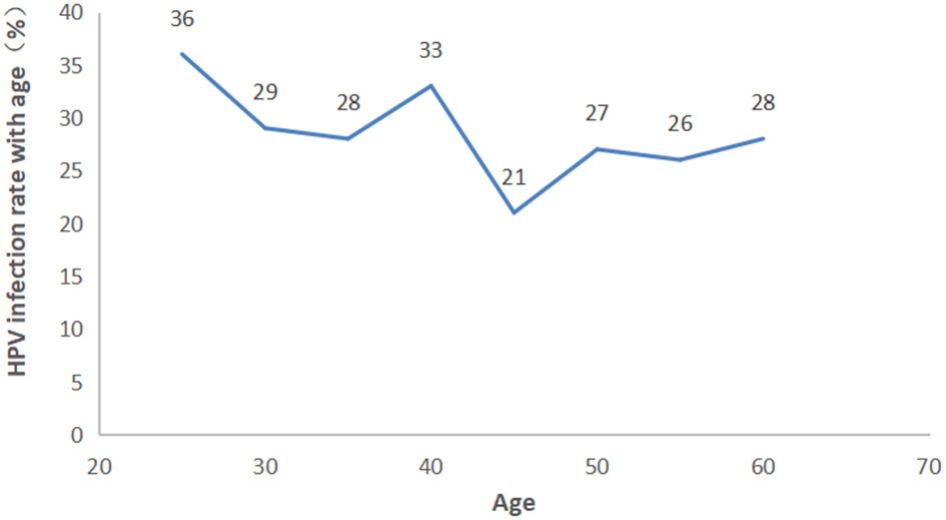
Prevalence of HPV grouped in each ae group.

The participants were categorized into eight age groups five years apart from 25 to 56 years. The HPV infection prevalence was 36.03, 29.06, 28.97, 33.68, 21.43, 27.60, 26.11, and 28.89% in each age group, respectively. As shown in Figure 4 and Supplementary Table 2, pure LR-HPV infection (including single and multiple LR-HPV infections) was the most prevalent, with 667 (18.08%) cases. Pure HR-HPV infections (including single and multiple HR-HPV infections) accounted for 187 (5.07%) cases. Mixed infections (including HR-HPV and LR-HPV mixed infection) accounted for 252 (6.83%) cases. In the different age groups, the number of men infected with pure-HR, pure-LR, or HR and LR HPV was significantly different (*p* < 0.001). Among the 1,106 men infected with HPV, there were two peaks; the first was 36.03% in the ≤ 25-year-old group, and the second was 33.68% in the 36–40-year-old group (Figure 5).

## Discussion

HPV infection in women has been widely studied worldwide. HR-HPV has been identified as the leading cause of cervical cancer, primarily in developing countries (8, 9). Characterization of HPV infection and the genotype distribution in men are serious clinical issues owing to the prevention of genital cancer in men and, consequently, HPV infection in women. Nonetheless, most studies on HPV infection in China have been conducted in women (10, 11), and data on the epidemiology of HPV infection in men are quite rare. Moreover, the HPV infection rate varies between nations and regions (6). In the present study, we assessed the prevalence and genotype distribution of genital HPV in sexually active men in Henan Province, located in central China. A widespread immunization program would influence HPV genotype distribution with HPV vaccines (12), but our study participants had never been vaccinated against HPV. Therefore, our results provide preliminary information on HPV genotype-specific prevalence in a high-risk cohort of sexually active men in Henan Province.

Thirty-two different HPV genotypes were detected in our study. The most common HPV genotypes were HPV 6 (24.88%) and HPV 11 (12.68%), consistent with some other reports (13–15). In line with several studies about the prevalence and genotype distribution of HPV in male genital warts (16–18), HPV 16 was confirmed to be the most frequent HR-HPV genotype and the third most frequent HPV genotype after HPV 6 and HPV 11 in this study. The results also placed HPV 18 and HPV 58 as the second most prevalent HR genotypes. Therefore, the vaccine-targeted HPV genotypes (HPV 6, HPV 11, HPV 16, HPV 58, and HPV 18) were among the most frequently detected HPV genotypes in our study. Potentially, the practicality of the 9-valent HPV vaccine may allow us to prevent the most frequent HPV genotypes in men in our region.

HPV 16, 58, 51, 39, and 52 have been identified as the most common HR-HPV genotypes causing male genital warts in Shanghai, but in the Guangdong province, the most common genotypes were HPV 52, 16, 81, and 58 (19, 20). In our study, the most common HR-HPV genotypes were HPV 16, 58, 18, and 39. We detected a high prevalence of vaccine-targeted HPV genotypes in our study, which is attributable to the fact that men in this region are typically not vaccinated against HPV. For example, the 4-valent or 9-valent HPV vaccines can prevent HPV 6, 11, and 16. The new 9-valent HPV vaccine does not cover HPV 84, 61, and 81; therefore, these cannot be prevented with vaccination. This indicates that a new HPV vaccine should be considered to cover the most prevalent HPV genotypes in this region.

Multiple HPV infection was associated with an increased risk of HPV persistence, though it was relatively low in the present study (21–23). The infection rate with multiple HPV genotypes was 12.20% of all cases. This rate was lower than that found by previous studies, which showed the infection rate with multiple HPV genotypes to be 56.7% (13), 33.8% (15) and 59.7% (24). The variation between the studies may be explained by differences in the detection protocols employed in different laboratories, the sampling approaches, and the geographical variations in HPV genotype distribution. The prevalence of HPV infection was the highest among men in the ≤ 25-year-old age group, and the difference is statistically significant between different age groups (*p* = 0.001, Supplementary Table 2), while several other studies showed there were few differences between different ages (25, 26). This study provided beneficial information about the epidemiology of genital HPV infection in men in the Henan Province, which should be utilized when evaluating the efficacy of HPV vaccines to prevent vaccine-targeted HPV genotypes. In conclusion, the prevalence of HPV infection was relatively high. Thirty-two different HPV genotypes were detected, and the most frequently detected genotypes were HPV 6, HPV 11, HPV 16, HPV 58, and HPV 18, respectively. We also found that an unmarried status and smoking increased single and LR-HPV infection. Multiple lifetime sex partners and not using a condom were more likely to cause LR-HPV infection. Understanding the epidemiological characteristics of HPV infection is essential to the development of prevention and control strategies for HPV.

In our study, even the 9-valent vaccine could not cover all the HPV genotypes that we most frequently detected. Although some HPV infections in young people are temporary and could be naturally cleared by the immune system, which will not result in clinical diseases, they may still lead to continuous virus transmission to their sexual partners. Therefore, it is necessary to monitor the infection status of HPV-positive men in our region.

## Data Availability Statement

The original contributions presented in the study are included in the article/Supplementary Material, further inquiries can be directed to the corresponding author/s.

## Ethics Statement

The studies involving human participants were reviewed and approved by the Ethics Committee of Henan Provincial People's Hospital. The patients/participants provided their written informed consent to participate in this study.

## Author Contributions

HW, YY, and GL: conceptualization. HW, YY, and WY: data curation. GL and XL: investigation. XL and JZ: methodology. HW project administration and resources. JZ: supervision. HW and YY: visualization, and writing and editing. All authors contributed to the article and approved the submitted version.

## Funding

This work was supported by the Joint Program of Medical science, Technology Research of Henan Province under Grant Number [LHGJ20190611 and SB201903018 to YY] and the Science Research Project of Henan Province (202102310355).

## Conflict of Interest

The authors declare that the research was conducted in the absence of any commercial or financial relationships that could be construed as a potential conflict of interest.

## Publisher's Note

All claims expressed in this article are solely those of the authors and do not necessarily represent those of their affiliated organizations, or those of the publisher, the editors and the reviewers. Any product that may be evaluated in this article, or claim that may be made by its manufacturer, is not guaranteed or endorsed by the publisher.
