# Supplementary material for: The Prevalence and Genotype Distribution of Human Papillomaviruses Among Men in Henan Province of China
Source: Front Med (Lausanne). 2021 Sep 20;8:676401. doi: 10.3389/fmed.2021.676401 (PMC8488141; doi:10.3389/fmed.2021.676401)
Supplement: Supplementary file 1 [file Data_Sheet_1.docx]

Table S1 Detection rate of HPV infection mode

| Mode | HPV-positive（n） | Detection rate (%) |
| --- | --- | --- |
| Single HPV infection | 656 | 17.78% |
| Double HPV infection | 139 | 3.77% |
| Triple HPV infection | 108 | 2.93% |
| Quadruple HPV infection | 89 | 2.41% |
| Quintet HPV infection | 64 | 1.73% |
| Sextuple HPV infection | 39 | 1.06% |
| Septuple HPV infection | 11 | 0.30% |
| Total | 1106 | 29.97 |
| p | 0.001 | |

Table S2 Prevalence of HPV infection with age

| Age | Total (%) | Single  (%) | Multiple  (％) | Pure-LR  (％) | Pure-HR  (％) | Mixed  (％) |
| --- | --- | --- | --- | --- | --- | --- |
| ≤25 | 680 (36.03) | 139 (20.44) | 106 (15.59) | 148(21.76) | 40(5.58) | 57(8.38) |
| 26-30 | 960 (29.06) | 155 (16.15) | 124 (12.92) | 164(17.08) | 49(5.10) | 66(6.88) |
| 31-35 | 870(28.97) | 165 (18.97) | 87 (10.00) | 156(17.93) | 39(4.48) | 57(6.55) |
| 36-40 | 380 (33.68) | 74 (19.47) | 54 (14.21) | 72(18.95) | 22(5.79) | 34(8.95) |
| 41-45 | 280 (21.43) | 37 (13.21) | 23 (8.21) | 37(13.21) | 12(4.29) | 11(3.93) |
| 46-50 | 250 (27.60) | 40 (16.00) | 29 (11.60) | 46(18.40) | 12(4.80) | 11(4.40) |
| 51-55 | 180(26.11) | 27 (15.00) | 20 (11.11) | 30(16.67) | 8(4.44) | 9(5.00) |
| ≥56 | 90 (28.89) | 19 (21.11) | 7 (7.78) | 14(15.56) | 5(4.56) | 7(7.78) |
| Total | 3690 | 656(17.78) | 450(12.20) | 667(18.08) | 187(5.07) | 252(6.83) |
| p | 0.001 | <0.001 | | <0.001 | | |
